# Phenomena of Cataphoresis

**Published:** 1897-11

**Authors:** Weston A. Price

**Affiliations:** Cleveland, O.


### Phenomena of Cataphoresis.

BY WESTON A. PRICE, D.D.S., CLEVELAND, O.

Abstract of paper read at American Dental Association, Old Point Comfort, Va. Aug., 1897.

In this paper I shall confine myself to the consideration of phenomena attending the application of an electric current to the human body, with and without an interposing medicament, and especially as applied to the dental organs.

There are three distinct theories as to the forces at work and their particular action in these processes, and there is such a diversity of nomenclature, and variety of methods for the application of these forces, that I shall include all the methods of applying an electric current to the dental organs for producing anesthesia, whether used in connection with a medicament, or not. These theories are: First. The polarization of the tissue producing an inhibition of the sensory impulse. Second. Osmosis. Third. Electrolysis.

The first theory provides that when an electric current is applied to a tooth, with or without a medicament, the conditions produced are almost or entirely due to the effects of the current and not to the medicament, other than as an assistance in conducting a current.

Prof. Neiswanger, of Chicago, has said that, owing to the liberation of hydrogen at the negative pole and of oxygen at the positive pole, there would be at these two regions, respectively, a condition of increased and decreased excitability, due to the production of an acid condition at the positive pole, and an alkaline condition at the negative pole, and that the zone of neutrality would be the median of resistance; and consequently that the negative or indifferent electrode should be placed as far as possible from the polarizing electrode. The adherents of this polarization theory maintain that the amount of current used is not great enough to produce sufficient electrolysis of the medicament used to produce its effect on the tissue.

The other division of the first theory is, that a constant current applied to the dental branches of the tri-facial nerve, with the positive pole applied to the tooth, and the negative pole over the gasserian ganglion, inhibits the normal sensory impulse. The leading advocates of this theory maintain that a certain and intricately definite amount of current applied in the manner just described will produce a condition of anesthesia in the tooth, and that either too little or too much will not produce this condition. They call it "short circuiting the nerve." For its application the positive pole of the constant current is attached to the dental engine in such a manner as to make the bur the electrode, the hand-piece being insulated, and the negative pole is applied over the gasserian ganglion, on the same side as the tooth to be operated upon, which is perfectly insulated. No medicament is used in either of these two processes except moisture in the former, the polarization theory to reduce the resistance of the dentine.

The next theory provides that the medicament, which is applied by the electrode, is the agent which does the work, but that it is carried in by a physical force. Dr. Morton has said, "You may even drive solid particles into tissue, and solid particles will move through fluids by the aid of electricity." He also says, "You cannot be too sure of your osmosis."

Dr. Peterson, in the International System of Electro-Therapeutics, says, "From a medical standpoint we understand by cataphoresis the introduction of medicaments by means of electricity into the body through the skin or mucous membranes. It seems to be a *purely physical process* and has nothing to do with electrolysis."

Electrolysis, the last theory, not generally accepted, provides practically that all the effect produced in passing an electric current through a medicament, as applied in cataphoresis or in any other way, is electrolytic.

Suppose the positive pole be placed in a tooth and the negative over the gasserian ganglion, and suppose that the sensory impulse is inhibited, just as the motor is.

WILL THE CURRENT TRAVEL ON THE NERVE OR ON THE SURROUNDING TISSUE?

To determine this I chloroformed a dog, and with expert assistance dissected out the inferior dental nerve from near the base of the skull to the inferior dental foramen and insulated. The inferior cuspid on the same side was excised and insulated and an electrode placed in the pulp tissue. With very great care the resistance through the path (pulp and nerve) was determined and found to be 23,630 ohms. The resistance through the path (the pulp to a point equi-distant away on the muscle), with the same electrodes, was determined and found to be 18,570 ohms, 5,060 ohms less than that of the nerve. This means simply that if the gasserian ganglion were right on the surface and the negative pole applied to it, the amount of current passing through each, the nerve and muscle, would be inversely in proportion to their resistance. But the nerve does not come to the surface, hence the current to pass through the nerve must travel along on it to a point opposite the negative electrode, and then pass out through the tissue to the surface, which the advocates of this theory claim it does. But an electric current always seeks the path of least resistance, so to fullfill the requirements of this theory the resistance through the nerve would have to be infinitely lower than the resistance of the surrounding tissue. A point of interest here is that the cross-section resistance of a nerve is still five times greater than its longitudinal resistance. The facts are that the current would diffuse throughout the entire tissue of that side of the face, and the amount of current flowing through the nerve at any point in cross-section would be less than that flowing through the same cross-sectional area of the tissue at any point around it, within a wide area. Hence the theory of *short-circuitiug* a nerve is inconsistent and its accomplishment in dental therapeutics clearly impossible. I have demonstrated this on the inferior maxillary of a sheep and on the sciatic nerve of a frog. This is a very important consideration, as it determines to a great extent the possibility of realization of the fundamentals of both the inhibition and the polarization theories. Now as regards

THE APPLICATION OF A CONSTANT CURRENT TO THE DENTAL ORGANS.

Where will the current leave the dental nerve if the positive pole be upon the exposed pulp and the negative on the cheek? Not to discuss the anatomical structure of the pulp—nearly two-thirds of a pulp consists of blood vessels and their contents; much less than one-third nerve fiber, and the balance connective tissue, etc. The blood has a greater conductivity than the nerve tissue for the same cross-sectional area, consequently most of the current will travel through the surrounding tissue and not through the nerve tissue. The size of the apical foramen and the resistance through the walls of the pulp-chamber are of some importance in this connection. In the case considered above but a very small percent of the entire current flowing would travel for any distance on the nerve fibers, since it would go direct to the less resistant tissue.. Suppose the current applied just as in the last case, except that the pulp is not exposed and the current must pass through the dentinal tubes. Here it seems to me would be an ideal condition for the demonstration of either the polarization or the purely inhibitory theory. We would expect that since these tubes contain chiefly the projected fibers of the adontoblastic or spindle-shaped neuclated cells surrounding the pulp, which are supposed to be the periphery endings of the dentinal nerves, the current would of necessity have to pass through this structure to the pulp before it could disseminate to the other structures. Of course the lime salts of the dentin are a non-conductor. If any effect is produced by applying the current in this manner through the drill it must be on this structure in the tubes, for it would disseminate immediately on arriving at the pulp tissue. These cell projections probably fill completely the space of these tubuli.

Will the current applied under these conditions produce anesthesia of the part to any considerable extent? I answer guardedly and after giving the question a very thorough investigation, No. From a physiological standpoint it would not be possible, since we are dealing with a sensory nerve, the most sensitive one in the body, whose function is to produce the sense of pain with the very slightest irritation. If the sensory nerve did not respond in the form of pain to the passage of the current, we would expect even then the amount of current neccessary to produce this condition, judging from motor nerves, would be many million amperes.

But you say the impulse which carries the sensation of pain in the sensory nerve is related to the internal nerve-current of the nerve, discovered by Du Bois-Reynold, and why could not this impulse be interposed by an artificial current, thus not permitting the pain impulse to pass? There are two answers: First. This nerve current always travels from center to periphery in motor nerves, and from periphery to center in sensory nerves, always in the direction of the impulse, hence it would be in the same direction as the polarizing current which really increases this nerve-current when in the same direction. We should then expect on his theory an increase of sensitivness. Again, the normal sensory impulse has no connection with this nerve-current, and furthermore, the origin of this nerve current would answer this question. I think it is inconsistent to expect to be able to pass enough current through the sensory nerve to produce that condition of anelectrotonus, since the pain limit will not permit of scarcely the thousandth part of so great a quantity of current to be passed as would be necessary to produce that same condition in a motor nerve, provided the sensory nerve were capable of an analogous reaction. For the clinical answer to this question I have tried in vain to produce this condition, using every variety of conditions in the tooth, and all combinations of potential and resistance possible in instruments on the plan of this theory. It is very simple and would be ideal if possible. Any good cataphoric apparatus will produce every possible combination of any of these instruments in use at the present time.

I have verified repeatedly that

THE RESISTANCE THROUGH A TOOTH, accordingly as the cavity is wet or dry, will vary from thousands to hundreds of thousands of ohms. I have measured cavities in dentin after dehydrating and found them to vary all the way from 20,000 to 1,000,000 ohms, and in different parts of the same cavity almost that amount of variation over the surface of the dentin alone, while through the enamel, of course, those figures will be multiplied by thousands.

Two things must be evident to everyone at a glance, viz., that in delivering the current to a tooth, from the bur as the positive electrode, it is impossible to have a uniform amount of current flowing as the bur is moved to different parts of the cavity, owing to the variableness of resistance of the different parts of the cavity; and that with so very high a resistance it would be impossible to have more than an extremely weak current flowing unless the potential were very many times that used.

With my instruments I can measure with precision and express in amperes any amount of current from a one-hundred-and-twenty-five millionth of an ampere to one-twentieth af an ampere. I measured the actual amount of current flowing in amperes in the use of two of these instruments, as used by their exponents, and found it to vary from six millionths of an ampere to one-twelfth millionth with one, and from one-sixth millionth to one-sixty millionth of an ampere with the other instrument. In view of the fact that this condition of anelectronus is in direct proportion to the amount of current flowing, I have passed as high as two milliampers of current through a healthy live pulp and tried to work on it at the same time, but could not produce this condition of anelectrotonus. I have even let it flow for fifteen minutes and not been able to touch the pulp even slightly without pain. We saw from the law established by Pfluger that this condition was in direct proportion to the amount of current flowing, hence how impossible for the millionth part of an ampere to produce this condition if two thousand times as much did not.

Another feature here appears, making the realization of the anelectrotonic condition of the dental nerves impossible, which is the law of stimulation of an electric current. This law holds that it is the sudden variations of current strength that excite muscular contraction in a motor nerve or in a sensory nerve produce its impulse, pain. Hence when the current is delivered to the tissue from a revolving bar, every blade brings with it an interruption of the current strength, and if the current strength were great enough to produce anelectrotonus it would cause excruciating pain.

### OSMOSIS.

This theory provides that when we apply an electric current to tissues, with an interposing layer of some medicament under the positive pole, that medicament itself will be carried into the tissue by means of a physical force possessed by the current. *What is osmosis?* It is the diffusion of a dissolved substance in a solvent to equalize the concentration.

From the few laws governing osmosis we can make some valuable deductions as to the role this force plays in cataphoresis. Since the nature of the solvent has nothing to do with the osmotic pressure, it at once becomes obvious that—First. It does not matter what solvent we use for our cocain (provided it is the force of osmosis that accomplishes the work). Second. Since the osmotic pressure is in direct proportion to the concentration, the solution should be as nearly saturated as possible. Third. Since the osmotic pressure is increased to a definite extent by each degree in increase of heat, the solution should be kept as hot as possible.

These hold good for practical application, if the force we are dependent upon is osmosis.

### WILL OSMOSIS CARRY COCAIN INTO DENTIN TO ANY CONSIDERABLE EXTENT?

To answer this I have sealed a saturated solution of cocain in cavities for two days, and again for two months, without producing anesthesia except on the very surface of the cavity. I have also applied it for some time on an exposed pulp and could not cut very far into it. Sulphate of strychnin and bichlorid of mercury applied on cotton to the chests of frogs produced no physiological effect, while with a current, death was produced in a few minutes. This answers the question whether osmosis alone can produce this condition. Now for the theoretical suggestions. We know that if a saturated solution of cocain be placed in a cavity there will be a difference of concentration between the cavity and dentin and the cavity and pulp. We know also that the velocity of migration of the dissolved salt will depend on two things: The osmotic pressure or "head," and on the resistance. We have all observed how slowly a fine precipitate settles. This is the resistance of the solution. Just so the resistance of any solvent to any dissolved substance can be determined from the osmotic pressure and velocity. Now as a matter of fact all cell tissue and porous partitions offer great resistance to osmosis, and many cells have the power of permitting some substance to pass while excluding others. In the dentine we have both of these conditions, and it would be expected that a very great barrier would exist here to the osmosis of the cocain. We are forced to conclude that osmotic pressure is not the force on which depends the transmission of cocain through dentin into the pulp. This brings us to a consideration of the last question, viz., What are the laws of electrolysis?

WHAT IS ELECTROLYSIS?

It is the change that is effected by the passage of an electric current in so far as the electricity exhibits itself as such. How is electricity conducted? As expressed by Nernst and translated by Palmer, "The conveyance of electricity in conducting substances may happen in two ways, viz., with or without the associated transportation of matter. The latter happens in the case of metallic conductors (first class); the former in electrolytic conductors (second class); hence these are called conductors of the (first) and (second) class respectively." The nature of conduction in the first class (metallic) is unknown, but the nature of conduction in the second class is perfectly understood. From the above author I quote that, "The process of the conduction of a current, as a result of electric forces in conductors of the second class, consists in the displacement of free ions. By free ions we mean those which are not united to each other to form electrically neutral molecules; the positive ions migrate in a direction from anode to cathode, the negative ions in the opposite direction."

A solution conducts electricity better the more numerous the ions and the smaller the friction which they encounter in their migration. This conception may be applied unchanged to every substance which conducts electrically—whether gaseous, liquid or solid; whether simple or a mixture. In so much as it is impossible for a weighable quantity of a substance to consist solely of positive or negative ions—because this would signify the accumulation of such immense quantities of electricity that the substance would at once be dissipated in consequence of the repulsion—only *compound substances* (but no elements) have the property of electrolytic conductivity. Moreover, the molecules of the conducting substances must be dissociated in order that there may be free ions present; *and the free ions are divided into two classes which are sharply contrasted*, according as they are positively or negatively charged. The electrolytic charges of the ions are equally great and equivalent whether they occur in solution or in substances having a simple composition. This would be anticipated, because the fundamental laws of Faraday hold good both for water solution and also for fused salts. It is very remarkable that *we do not know of any electrolyte which, in the pure state and at ordinary temperatures, has the power of (electrolytic) conductivity to any marked extent.* Thus, liquid hydrochloric acid or pure water cannot conduct electricity noticeably. But when they are mixed they become conductors. The reason for this certainly is not that the ions in the pure liquid experience too great a resistance to their movement, but rather that the liquid electrolytes in the pure state are dissociated only to the very slightest extent. So, in the passage of a current through any liquid except a metal, the current can pass only by dissociating some of the molecules, called the ionization of the medium, and these ions which are equal in quantity migrate in their respective directions. The measure of the ions traveling in either direction is the exact measure of the ions traveling in the other direction and the measure of the current flowing.

This is expressed by Nernst as follows—" When the galvanic current passes through conductors of the second class, viz., electrolytes, then, in addition to liberation of the heat, there occurs a transportation of matter (migrations of the ions); and also on the limiting surfaces between the conductors of the first and second classes there occur peculiar chemical processes, which latter consist primarily in the solution of the electrodes or in the separation of the ions from the electrolytes, but they are usually complicated by secondary reactions between the electrolyte and the separated products." This reaction and the phenomena attending it has, by a new reaction of this ion in a different place, forming again an insoluble precipitate, been taken by some as a proof of the movement of solid substance through the solution.

The quantity of the ion separated in unit of time upon the electrode is proportional to the intensity (strength) of the current; and the same quantity of electricity will in the most differing electrolytes electrolyse chemically equivalent quantities of ions. In those cases where the chemical value of the ions is capable of changing, of course the meaning of *chemical equivalence* changes; thus the same current which separates 200 grams of mercury from a solution of $HgNO_3$ will separate 100 grams from a solution of $HgCn_2$. The above author says, "It is only in the rarest cases that the ions themselves are formed as the products of electrolysis, i. e., products which have the same composition as the ions as they are primarily separated, which differ from the ions only by having given up their electric charges. Thus the hydrogen ions appear in the form of $H_2$; the ions of certain metals in the form of metals; and under suitable conditions the ions of the haloids (acidiferous elements) in the form of metaloids. But much more frequently the ions either act upon each other on being separated, as in the decomposition of the acetic acid ion according to the equation, $2CH_2COO=C_2H_6+2CO_2$, producing ethene and carbon dioxid; or the ions may react upon the water, as when separated sodium gives off secondary hydrogen; or finally the ions may react *upon the metal of the electrode*, as when separated chlorin forms the respective clorid."

The (freshly separated) ions, which have been deprived of their charges by aid of the passage of powerful electric energy, are illustrations of substances containing a large quantity of free energy, that is, illustrations of great affinity. The (freshly separated) ions are able to perform reaction of which they are quite incapable in the ordinary state; thus freshly separated hydrogen, unlike ordinary hydrogen, can reduce nitric acid to the so-called "nascent state. It is especially worthy of our notice here that electrolytic dissociation, as compared with ordinary dissociation, is influenced but slightly by temperature. In fact it sometimes happens that with rising temperature it diminishes, or it may slightly increase, which is in strong contrast with ordinary dissociation, which always rapidly increases with rise of temperature.

Let us now apply some of these laws of electrolysis to the particular process in which we are interested. Suppose the positive pole to be applied to the dentin of a tooth and the negative to the cheek. An interposing layer of medicament, say cocain in water solution, is between the metallic positive electrode and the dentine. Now the only way electricity can get from the metallic positive electrode to the metallic negative electrode is by the dissociation of some of the molecules in every substance of the second class through which it passes. In every part of the course through the cocain solution, the dentine, the pulp, the connective tissue, the blood vessels, the muscle tissue, and sponge on the negative electrode, there will be a cleavage of some of the molecules of the various chemical compositions into a positive and a negative ion. These ions, with equal force and chemical equivalence, start on their respective journeys toward their opposite poles. They meet with friction, which varies for different ions, and since they have the same forces behind pushing them, their velocities will vary with their resistance. If in their course they meet a new ion or an element or a compound for which they have a greater affinity than the force which separated them, they will unite with it until they are again called into service. Unless an ion found such an affinity it would keep on going until it got to the metal plate of negative electrode, and if it could unite with it would do so. If not, would be deposited upon it or be liberated in the form of gas.

Now the question we have raised so often,

"IS THE CURRENT WE USE STRONG ENOUGH TO PRODUCE ANY ELECTROLYSIS?"

must be settled, for if we have no electrolysis we cannot have any current flowing, and the measure of the current is the measure of the electrolysis, and vice versa. In the case above the hydrochlorate of cocain would be broken up. The positively charged ions passing into the tissue toward the negative pole will doubtless meet negatively charged ions for which they may have great affinity coming towards the positive pole, and at once in the tissue there will be formed a new product. Now it is a fact that only a small percent of the molecules will ionize or dissociate, hence we see why there has been practically no difference in the effect we observed from using a saturated solution of cocain or a one percent solution.

I have observed the following results by applying the cocain solution as stated above: *First.* In nearly 1,500 cases, of which I have kept a record, the percent of perfectly successful operations has been between 95 and 100, and of late, with the increase of experience, all cases have been successful on the second application, if not on the first. Of these fully 100 were cases in which the pulp was entirely removed at the time, and in about 200 more the pulp was drilled into and partly removed and a devitalizing agent used to complete the destruction. *Second.* In almost all cases of single-rooted teeth the pulp was entirely removed at the time. *Third.* No sensation of pain was felt from the devitalizing agent applied after using cataphoresis. *Fourth.* Not a single case as yet of a dead pulp from the use of cataphoresis. *Fifth.* No difference has been noted in the time required for varying concentrations of cocain or any other agent. *Sixth.* The average time required for all cases was about thirteen minutes. *Seventh.* The amount of current tolerable is determined by the effect of the cocain on the pulp tissue, and not in the dentine, in a case of unexposed healthy pulp. *Eighth.* The resistance through the wet dentine varies all the way from a few thousand to five hundred thousand ohms. *Ninth.* The amount of current tolerable has been found to vary from one two-hundred thousandth to two thousandths of an ampere, the average being less than two ten-thousandths at the beginning of the operation and four ten-thousandths at the finish. Of course, where pulps were devitalized a very much stronger current was used for the finish, though seldom more than that amount was used where the continued life of a pulp was expected. *Tenth.* There are constant symptons that will give any indication of the amount of current flowing. Each case has a different pain limit. Hence the absolute necessity of using a milliamperemeter. *Eleventh.* No effect has ever been noted in the tissues beyond the tooth, except where a very strong current was used, then slight periostitis.

WHAT DEDUCTIONS CAN WE MAKE FROM THESE RESULTS?

We know that when a solution of cocain is applied to the dentine of a tooth, under the positive pole, with a negative elsewhere, we do get anesthesia, not only of the dentine, but of the pulp tissue, if applied long enough. Will the current alone produce this condition? No. Then it is not the current that does the work. Will the medicine alone produce this condition? No. Then it is not a simple osmosis: Does the current so applied produce any change in the cocain solution? Yes, it cannot pass through it except by changing it.

Is there a transmission of matter in this solution under these conditions by simple osmosis? Probably very slight. Is there a considerable transmission of matter at all? Yes, by the migration of the ions. Do these ions produce currents which carry the unchanged medicine with them? Theoretically yes, but practically very slight. Are these currents produced in both directions? Yes. Can an osmosis be produced from a negative to a positive pole? Yes in some solutions.

According to the older theories there was supposed to be a physical force exerted in some manner by the electric current applied in this manner, and this idea has its advocates yet. There is a new theory, however, which is made by its introducer, Nernst, of Gottingen, Germany, to explain all the phenomena. He thinks that there is no transmission of matter except the ions themselves. Now to follow this argument in this connection, if it is the force of osmosis on which we are dependent, what will be the effect of new molecules of hydrochlorate of cocain being formed in the pulp tissue by electrolysis on the osmosis from without? Of course, if such be formed, it would diminish the difference of concentration within and without the pulp chamber, and by doing so would lessen the force of osmotic pressure, which is in direct proportion to the difference of concentration at various points. In this case it would not seem that the current was helping the process. It is certain that we are not dependent on a difference of concentration for the development of the force that carries the cocain through the tissue.

DO WE KNOW THAT A MEDICAMENT CAN ACTUALLY BE FORCED INTO THE PULP AT ALL?

Yes, in many ways. I have put sulphate of morphin with the cocain, and after extracting the pulp on a broach been able to detect the morphin by the nitric acid test under the microscope. I have killed a frog in twenty minutes with sulphate of strychnin with the current, when neither the current alone nor the medicine alone, left for a considerable time, produced any noticeable effect. I do not believe the medicine was in any case carried in as the original chemical species, but was charged by electrolysis. And further, with the conditions under which we use cataphoresis, I believe the forces upon which we are dependent are the dissociation of the molecules and the increased energy of these dissociated products. These ions by their migration and by the new chemical species they form, are capable of producing perfectly analogous phenomena under different circumstances, but under analogous conditions. The final goal is of course

TO DIMINISH THE TIME.

I do not believe this will be done by seeking directly for a substance that has a high osmotic pressure, but rather in seeking for a reaction that will produce the most active ion It is true, however, that substances which have a high osmotic pressure have good conductivity. Since the amount of current we can use is limited by the pain limit of the tissue, and the amount of electrolysis is a constant expression of the strength of current, of course the amount of chemical energy we can liberate is fixed, and we can not hope to change this unless we can change some of the laws of either *physiology, electrolysis or chemistry.* We have left these unfixed conditions to modify, viz., to select the ions with greatest migration velocity and which themselves or the compounds they will form will produce greater physiological effect upon the tissue for the same unit of concentration in the tissue. There is no reason why great advancement should not be expected in this direction, and it is my opinion that when it does come, it must come along this line.

